# How trade can drive inclusive and sustainable food system outcomes in food deficit low-income countries

**DOI:** 10.1007/s12571-021-01218-z

**Published:** 2021-10-13

**Authors:** Siemen van Berkum

**Affiliations:** Wageningen Economic Research, part of Wageningen University & Research (WUR), Pr. Beatrixlaan 582 - 528, 2595 BM Den Haag, The Netherlands

**Keywords:** Trade, Competitiveness, Diversification, Food security, Externalities, Trade-offs, Policies

## Abstract

Recent decades have seen food markets and value chains become increasingly global—a trend that creates challenges as well as opportunities for food systems. Positive trade effects on food security are not always self-evident in food deficit low-income countries. Moreover, whereas international trade may also be used to balance regional differences in climate change impacts and biodiversity, trade can exacerbate environmental challenges associated with food production, land use and climate change. This article argues that, for trade to drive inclusive and sustainable growth of nutritious food production in food deficit low-income countries, policies and investments in these countries must focus on three key priorities: 1) diversifying production and markets to increase resilience to external shocks; 2) enhancing competitiveness and improving market access for local farmers and SMEs, and 3) incorporating externalities in international trade. The latter requires collective international action.

## Introduction

All countries import food, but trade does not automatically enhance food security for all – particularly for the most vulnerable populations in developing countries (IPES Food, 2017; OECD, 2019a, b; IISD, 2019). The recent global spread of the coronavirus (COVID-19) and its disruptive consequences for food security has illustrated again how vulnerable internationally connected food value chains can be (IFPRI, 2020). Moreover, trade can exacerbate environmental challenges associated with food production, land use and climate change (Bellmann et al., 2019; Brown et al., 2017; Balogh & Jambor, 2020), although international trade may also be used to balance regional differences in climate change impacts, water availability and biodiversity, and is increasingly regarded as a potential adaptation mechanism (e.g. Janssens et al., 2020; Balogh & Jambor, 2020). This paper argues that with trade-compliant domestic policies that support sustainable and inclusive value chains, low-income food deficit countries can strengthen the competitiveness of their food and agricultural sector and enhance national food security. In addition, to support environmentally sustainable, nutritional, safe and inclusive food systems, countries should jointly pursue trade agreements that reinforce non-market values, such as food safety, environmental quality or nutritional content, as well as decent labour conditions.

This article uses a food system approach (Béné et al., 2019; HLPE, 2017; Ingram, 2011) to analyse implications of trade in agricultural and food products for food system outcomes, and brings forward several suggestions for trade-related policies and investments to counter potential trade-offs among social, economic and environmental objectives aiming at achieving SDGs. First, in Sect. 2, the benefits and potential trade-offs of trade are discussed. Next, Sect. 3 presents some global trends in and drivers of international trade, followed by Sect. 4 showing the increased food import dependency of low-income countries. Section 5 points at diversification of production and markets as strategy for low-income food deficit countries to build resilience to external shocks. Another strategy to external shocks on food markets is to strengthen competitiveness of the domestic agricultural sector. Section 6 discusses trade-compliant policies that are crucial in this regard. The role of standards in international trade is highlighted in Sect. 7, showing how small-scale farmers can be supported to participate in competitive value chains. Section 8 makes the point that to support environmentally sustainable, nutritional, safe and inclusive food systems, countries should pursue trade agreements that reinforce these non-market values. Section 9 concludes.

## The role of trade and policies in providing food security

There is much historical evidence that international trade promotes economic growth, as it allows countries to use its resources more efficiently by specializing in products and services it can produce most competitively (e.g. Brooks & Matthews, 2015; Martin & Laborde, 2018). Economic growth is assumed to directly contribute to poverty reduction, as it creates employment opportunities and reduces prices, among others for food, from which all – also the less affluent – consumers can benefit. Following this argument, there is a positive association of international trade with all four dimensions of food security:

- Trade contributes to food *availability* by enabling imports to cover shortfalls in domestic supply, thus contributing substantially to meeting food demand in food deficit countries.
- Trade increases food *access* by speeding economic growth—which boosts incomes and food purchasing power—and by lowering consumer prices.
- Trade improves *food utilization* because of greater overall demand for food (due to economic growth and higher incomes), and because a larger income share can be devoted to the purchase of nutrient-rich food. In addition, trade may contribute to a more diversified diet by providing various food products otherwise not available locally.
- Trade increases food *stability* by balancing food surpluses and deficits, by reducing seasonal effects on local food availability and by making local markets less prone to policy or weather shocks.

Despite the widely acknowledged links between increased trade and improved food security, trade can also pose numerous challenges to food systems in low income food-deficit countries. In these countries, increased trade brings a risk of higher dependence on food imports. This puts local producers under growing competitive pressure—and it makes consumers more vulnerable to external shocks in food availability e.g. (Koning & Pinstrup-Anderson, 2007; De Schutter, 2011; Mary, 2019). Another concern is that increased access to cheaper, more diversified food through open trade may not necessarily improve the nutritional quality of diets. By supporting the ‘nutritional transition’, trade openness can also increase access to unhealthy food and thus drive overweight and obesity (Global Panel, 2020). Further, recent international price spikes—in 2007/2008 and 2011/2012—have cast into doubt the assumption that trade openness makes food markets more stable – while market became more unstable as net exporting countries declared export restrictions (Morrison & Sarris, 2016). The recent global spread of the coronavirus (COVID-19) and its disruptive consequences for food security has illustrated again how vulnerable internationally connected food value chains can be (IFPRI, 2020).

**Fig. 1:**
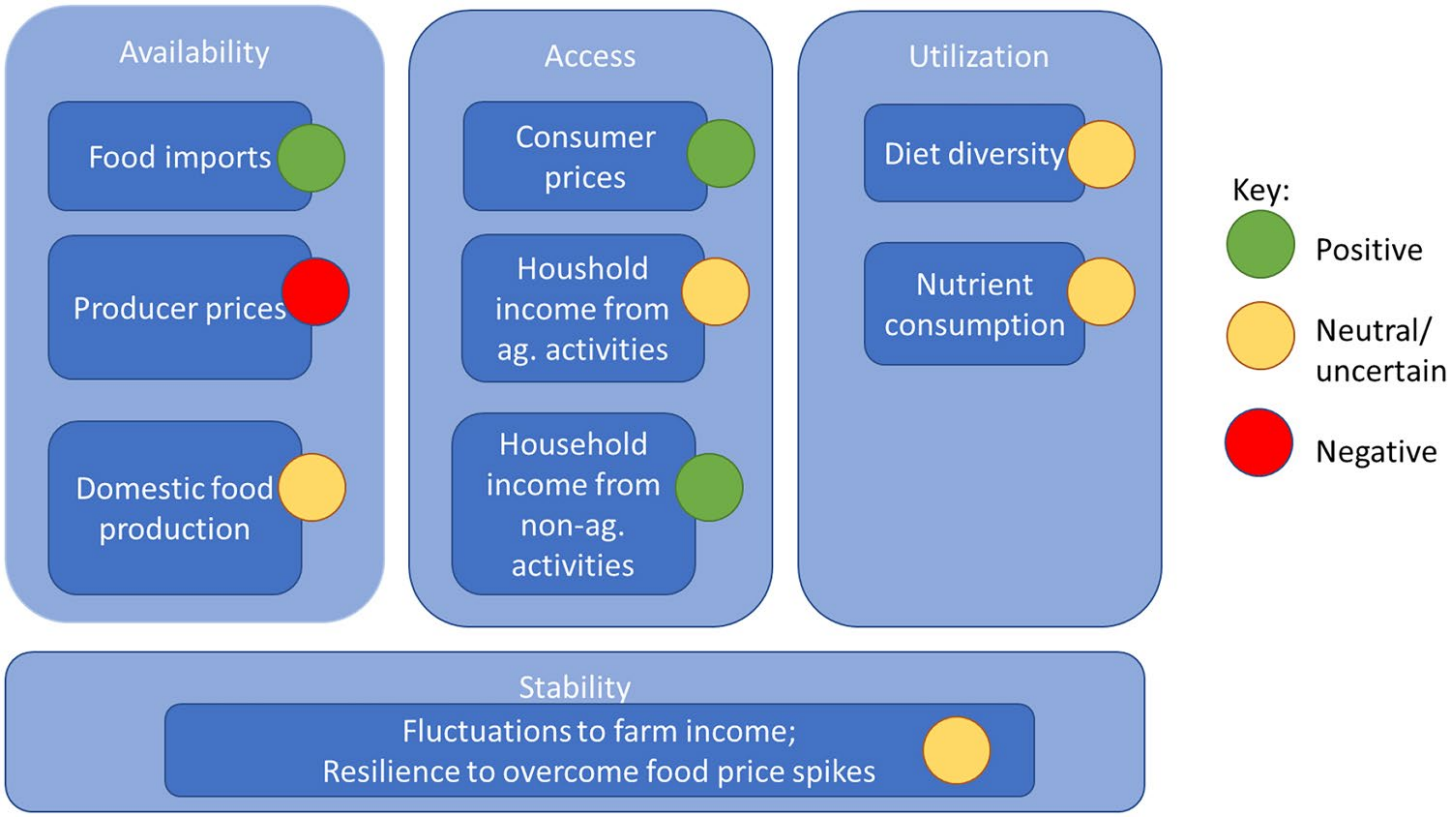
How international trade affects the four dimensions of food security in food deficit countries

Because of these concerns, the net food system impact of international trade—and of policies to boost trade even further—is uncertain. The effects of trade on various dimensions of food system security can be positive, negative, or neutral, can also be different for each segment of the society (e.g. food producer, consumer, trader, non-agricultural activities), and also context-specific (Fig. 1). International trade thus comes with complex trade-offs that need to be addressed through a decisive package of policies.

It is also clear that next to trade, food security is much affected by macroeconomic factors (Diaz-Bonilla, 2015; Brooks & Matthews, 2015; OECD, 2019a, b). Indeed, macroeconomic factors influence the four components of food security through different channels. Domestic production and imports determine availability (first component), and economic growth, generating employment opportunities and higher income levels, is strongly linked to food access (second component). In fact, it is evident that the ultimate driving force of global food security is the overall level of economic development, affecting each of its dimensions (Timmer, 2002; Regmi & Meade, 2013). Government revenues might also be used to implement policies and investments in favor of food security such as research and development (affecting availability and stability, the first and fourth component of food security), basic health services and food assistance and social protection programs (affecting use/nutrition, the third component). Nutrient security pertains to the individual the most, but is largely affected by income and access to food determining factors (e.g. Global Panel, 2017). From this perspective, actions that affect non-agricultural markets and employment - such as building infrastructure or ensuring equitable access to education – could be just as important for food and nutrition security as policies and investments in the agri-food sector. On the whole, this means that the discussion on trade and food security needs to be placed in the context of an overall framework of macroeconomic and exchange rate policies (Diaz-Bonilla, 2015; OECD, 2019a, b).

## Trends in international trade of food and agricultural commodities

**Fig. 2:**
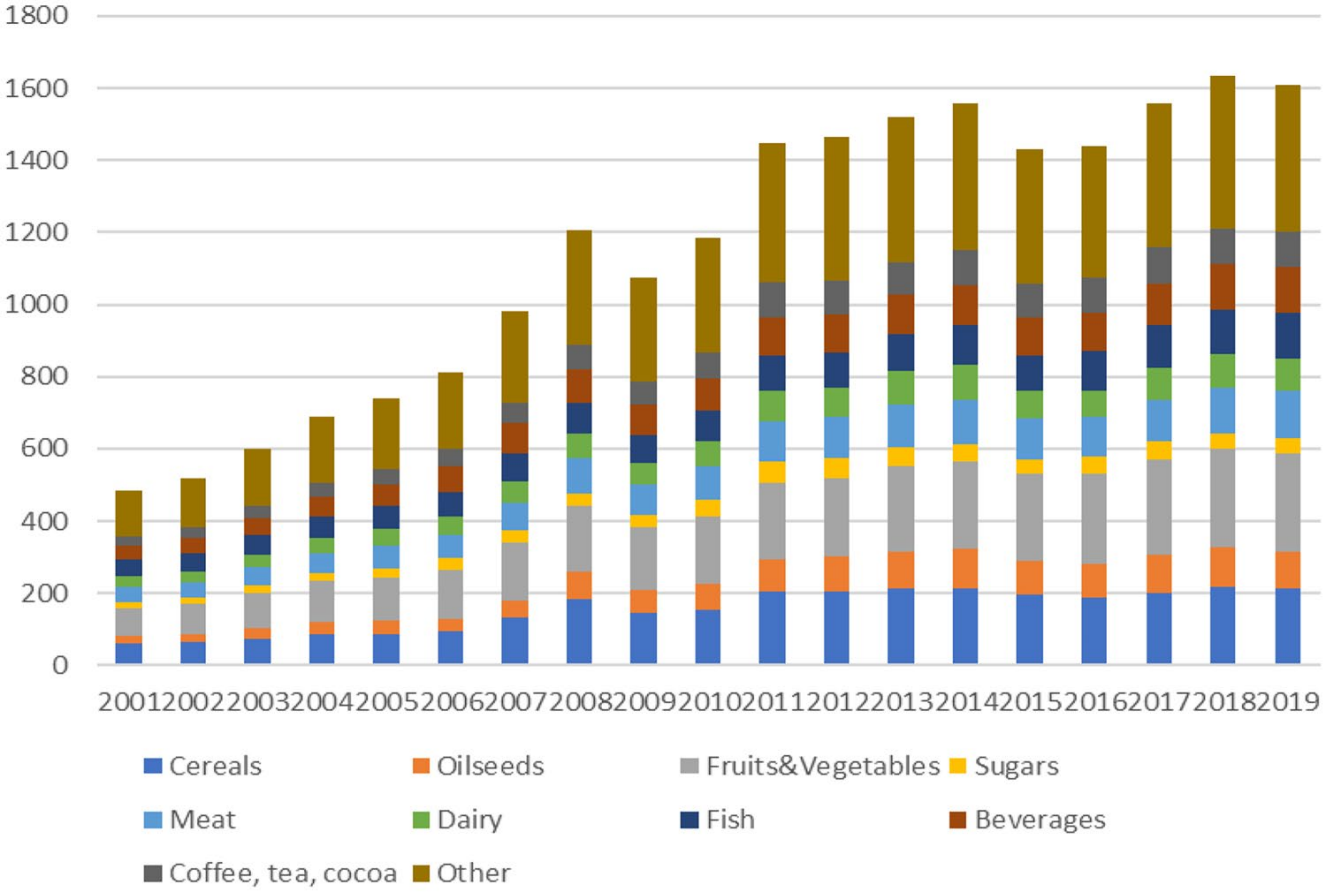
Development of global trade in agri-food products (world total imports, bn US$). Source: ITC

During the past half century in which agricultural production has trebled globally, trade in agricultural commodities and food products increased eight-fold, with an acceleration in growth in the most recent two decades (Fig. 2). Even while the majority of food produced around the world is used domestically, trade increasingly contributes to feeding the world’s population.

In value terms – note that all prices and values in this article are nominal unless explicitly stated otherwise - international trade in food and agricultural products has increased from almost US$500 bn in 2001 to over US$1610 bn in 2019 (ITC, 2020). Growth in global trade in value has been fastest in products such as oilseeds, fruits & vegetables, meat and fish, rather than in staple grains which nevertheless continue to dominate food trade in absolute volumes (Global Panel, 2020). These increases reflect demand from expanding populations, greater demand for diversified diets as incomes rise and a shift in diet particularly in many middle income countries towards more animal and processed products. For example, growth in trade in oilseeds has primarily been driven by demand for livestock feed, particularly from China which is currently recipient of around two-third of all global soybean imports. Oilseeds crops are also used in many ultra-processed product foods, global sales of which have increased dramatically since the early 2000s particularly in low and middle-income countries (Vandevijvere et al., 2019). Increased trade in sugar and sweetener products is also associated with a rapid growth of sales of sugar-sweetened beverages in many developing countries, most significantly in Latin America & Caribbean and in South and Southeast Asia. Demand for variety, convenience and year-round availability has been a driving factor after the rapid growth of global trade in fruits (with bananas, apples and oranges as most traded products) and fruit and vegetable product (Huang & Calvin, 2012; Rabobank, 2018).

Strong growth in trade in agri-food products has been supported by trade and investment liberalisation policies and rapid economic growth in China and other emerging economies (e.g. OECD, 2019a). Falling tariffs and reductions of trade distorting producer support have added to the gains in market access that began with countries implementing their commitments under the GATT Uruguay Round 1994 Agreement on Agriculture. In the last decades, applied average import tariff rates have declined further largely because of a range of bilateral and regional trading agreements coming into force and unilateral actions by some countries^1^ (OECD, 2019a). This shift in protective trade policies has had an important impact on production and trade patterns in the last two decades, with an increasing relative importance of production centres towards emerging and developing regions, those of Asia and South America in particular, and, in contrast, modest production growth in the developed production regions of Europe and North America. Consequently, developing and emerging countries are rising in importance as major agro-food exporters and importers – Brazil, the Russian Federation, India, Indonesia, China and South Africa in particular. Between 2000 and 2016, low- and middle-income countries’ share of world agricultural exports increased from 29% to 39%, while their share of world agricultural imports increased from 21% to 32%. There has also been a change in the distribution of trade between countries, with an increase in trade between emerging and developing countries, which implies an expanding South-South agricultural trade (OECD, 2019a; FAO, 2018).

## Increased food import dependency of low-income countries

**Fig. 3:**
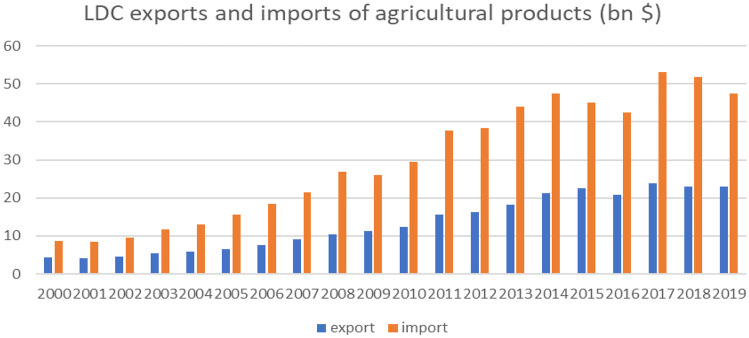
LDC exports and imports of agricultural products, 2000-2019 (in bn$). Source: FAO FAOSTAT data on crops and livestock products trade

The least developed countries (LDCs), as a group, increasingly depend on food imports. Over the past two decades, their combined annual imports of agricultural and food products have risen more than fivefold—from $8.7 billion in 2000, to around $50 billion in 2017–19 (FAOstat). As exports have risen more slowly, the LDCs’ joint agricultural product trade deficit has substantially increased: since 2011 it has exceeded $20 billion, and it reached $29 billion in 2017–18 before falling back to $23 billion in 2019 (Fig. 3). The increase of food imports results from rapid population growth rather than income growth. In most LDCs agricultural productivity growth could not keep pace with population growth, which is the reason why many countries in this group saw a rapidly increasing food import bill over the last two decades.

**Fig. 4:**
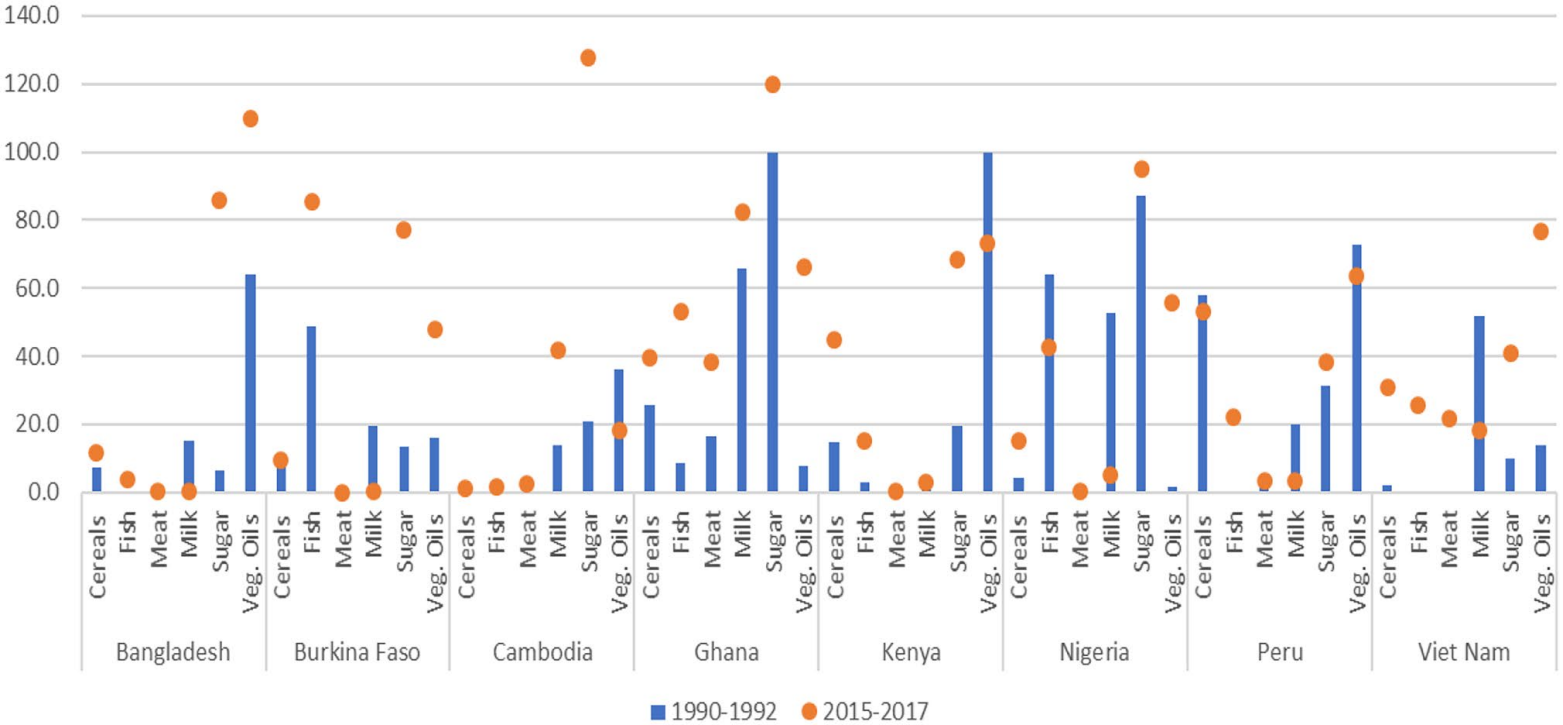
Share of imports in domestic food supply (in kcal/capita/day) in selected low and middle income countries (LMICs). *Source:* FAO Food Balance Sheets. *Note:* FBS import in tonnes is converted to kcal/capita/day based on the ratio between FBS food supply in tonnes and FBS food supply in kcal/capita/day. For some products, percentages are above 100, which means that production (and stocks) are very low and the country mainly imports this product, yet there is also some exports which brings domestic supply available below the level of imports

For a number of low-income countries, rising imports have led to higher import dependency over the last three decades. But because markets for different products are changing in various directions, countries face a range of net-trade positions and food import-dependencies which evolve differently over time. These more complex dynamics do not appear in the regionally aggregated totals of Fig. 3. To illustrate this for a number of countries their import dependency positions for several product group over times are displayed in Fig. 4 (see also AGRA, 2020; on country and regional developments in Africa). Figure 4 shows shares of imports in domestic supply for six important food categories in the early 1990s (blue columns) and compares these shares with those in 2015-2017 (orange dots). Results for the presented countries show import dependency is increasing for the vast majority of product categories.

Regions and countries with both high import reliance and low domestic food availability face specific challenges to their food stability. High import dependency easily creates food security risks, in the case of crop failures in foreign suppliers and/or policy changes that can cause supplies and international prices to fluctuate. The chances of supply disruption are further increased if the importing country depends on just one or two suppliers—often the case with commodities such as wheat, rice, palm oil and soybean, where the concentration of exporters is high (ITC, 2020; OECD-FAO, 2019). Diversification of supply sources is an important additional strategy for reducing risks to food security (Kummu et al., 2020).

Food import dependency becomes a severe problem when countries are less able to finance food imports—a risk that is highest if a country’s economy depends heavily on commodity exports and/or imports. A study of 129 low and middle income countries (LMICs) shows that high export and import dependence on primary commodities had a statistically significant and negative effect on food security over the 1995–2017 period (FAO, IFAD, UNICEF, WFP and WHO, 2019). Moreover, 80 percent of all the countries that saw a rise in hunger during recent economic slowdowns have economies that are highly dependent on primary export or import commodities (or both).

## Diversifying production and markets for more resilience

Food-deficit developing countries that depend heavily on export commodities (such as coffee, cocoa, tea, palm oil or rice) may face food security risks from a deterioration in those products’ terms of trade. It is thus vital to promote commodity and market diversification (Newfarmer et al., 2009; Diao et al., 2012; McIntire et al., 2018; Mania & Rieber, 2019). Otherwise, if trade dependency is mainly related to import demand, diversification of domestic food production is required. These structural transformations should be pro-poor and inclusive. Based on an extensive analysis of export diversification options in Mali, Chad, Niger and Guinea, López-Cálix (2020) identifies several key elements for simultaneously reinforcing market infrastructure and market exchange conditions. This requires targeted investments in market infrastructure (for efficient logistics) and in human capital (for building skills that enhance people’s productivity and employability), as well as government interventions that reduce specific institutional deficiencies, such as a lack of information and knowledge about market standards.

In addition to structural adjustments on the supply side, opportunities on the demand side in domestic and foreign markets should be exploited to achieve the transition to a more productive and differentiated agriculture, improve access to food domestically and create jobs outside agriculture. Strengthening regional trade relations with more or less equally developed neighbouring countries is a way to leverage trade to make the transition to a more diversified economy. By focusing on regional trade, countries can further exploit and develop their comparative advantages on nearby markets, using the generally existing social connections through which local consumers tastes are understood, and that can be served by short chains in which small-scale farmers and traders can participate.

Across Africa, promising opportunities exist for boosting intra-regional trade in food, agricultural and industrial products and services (World Bank, 2012; ODI/World Bank, 2013; Morrison, 2016; AGRA, 2019, Andam et al., 2019). Generally, regional trade agreements and market integration strategies can be an engine of growth, as shown in Europe, North America and Southeast Asia. Yet regional trade within the Africa region is still fairly limited: less than 20% of all exports. One reason may be that existing regional trade agreements, such as the Common Market for Eastern and Southern Africa (COMESA), the East African Community (EAC), and the Southern Africa Development Community (SADC), frequently exclude free trade in foods, since their product portfolio is rather similar and countries consider each other as competitors..

As food demand in Africa rises with population and income growth and - as diets become more diverse with rising incomes and urbanization—new initiatives to reduce intra-regional trade barriers show important economic potential. The recently established Africa Continental Free Trade Agreement (AfCFTA) may stimulate intra-Africa trade, accelerate export diversification and diversify export destinations and types of goods produced in the region (Brookings, 2019). In particular, the AfCFTA promises to increase intra-regional trade in food products that, if accompanied with the right measures, can greatly help boost smallholder farmers’ productivity growth and prospects for integrating into food value chains (UNECA, 2018; AGRA, 2020). To make the most of these opportunities, governments will need to reduce transaction costs by improving trade facilitation —such as import customs clearance procedures and port handling at the border—and invest in physical infrastructure, including roads, rail tracks and harbour facilities.

## Enhancing competitiveness with trade-compliant policies

One strategy for increasing resilience to external food market shocks, is to strengthen the competitiveness of the domestic agriculture and food sectors by increasing productivity. Higher productivity levels determine farmers’ ability to increase and sustain higher incomes, and also to supply food at lower prices to consumers. Several aspects are crucial in this regard. Markets must function properly with low barriers to entry and reduced risks. Market prices and margins should permit smallholder to remain actively involved in trade. Trade policy instruments (such as tariffs and other trade-facilitating measures) must be conducive to smallholder farmers to participate in the market and become part of modern supply chains. Supportive policies should be in place to guarantee that market engagement also leads to welfare improvements. However, poor countries have far less opportunities and limited resources to engage in market competition or trade facilitation policies.

### Ensure competition in agricultural markets

Competition in food and agricultural markets is a crucial dimension of food security: the degree of such competition determines the possibilities for smallholder farmers’ participation in food value chains and markets. Therefore, governments pursue competition and market entry policies to support the position of farmers and middlemen in domestic food value chains, to safeguard the public interest in food security, and to promote a more equitable distribution of wealth.

The degree of competition in agriculture markets has large implications for the formation of prices and the distribution of rents along the value chains. It may provide incentives for modernization and investments by smallholders, and it shapes the space for value chain interventions to support poor (but efficient) producers. Conversely, a lack of competition can lead to local monopoly rents that substantially reduce the welfare of consumers, the income of farmers, and the effectiveness of government policies (FAO, 2018; Mooney, 2018; Bellmann et al., 2019).

Generally, market configurations and competitiveness vary considerably within and across countries and regions. Despite pervasive concerns about competition in food and agricultural markets in developing countries, little empirical evidence exists for non-competitive pricing (see, e.g. FAO, 2018; OECD, 2019a, b; Deconink, 2020). Focusing on grain markets in Sub-Saharan Africa, Dillon and Dambro (2017) find that food markets in these countries are generally quite competitive. However, illustrated by a case study in the Indonesian dairy sector, Treurniet (2020) claims that concentration of traders or processors easily occurs in markets for perishable foods where transaction costs are high and climate conditioned transport is vital for quality compliance.

Because agriculture is at the base of a food value chain that further includes processing and retailing, market power may exist at either or both of these stages. Market power can be difficult to measure, because of conceptual and data issues (e.g. Sexton and Xia, 2018), but export firms have many ways to charge non-competitive rents—especially when markets are concentrated at the global level, as with cocoa grinding and coffee and banana export (Lee et al., 2012; Anania, 2015)

**Fig. 5:**
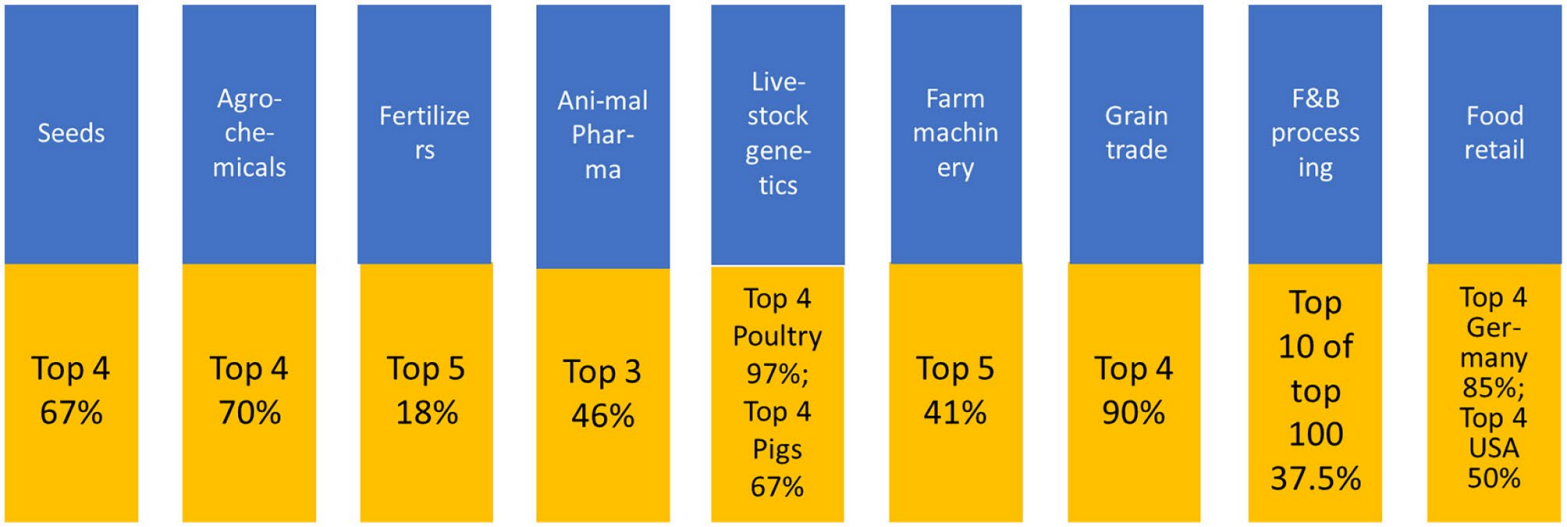
Worldwide market shares of the largest companies in the agricultural and food sector. Source: composed and adapted from IPES Food (2017) and Mooney (2018)

Figure 5 illustrates the current concentrated food market structure. The division of the supply chain into segments gives a somewhat distorted picture, because companies (conglomerates) are active in several parts of the food chain For instance, four transnational companies have an estimated two-third of the global market share in seeds. Three of them have also the biggest stake in the globally operating chemical inputs (pesticides) industry, in which the top-5 control 70% of the global market (Mooney, 2018).

In sum, food security policies that target farmers or consumers, and that rely on trade policies, need to reflect the extent of competition throughout the supply chain and the bargaining power between the relevant stakeholders. Such policies also need to account for a dialogue with local and globally operating food firms. In other words—over and above the investments in food market infrastructure and knowledge outlined earlier—trade policies require inclusive governance regimes as organisation-like entities, simply to balance interests among key parties.

### Use WTO compliant policies to make domestic agriculture more efficient and competitive

A food net-importing country could build their own domestic production in order to improve domestic food availability. However, if this would imply imposing import restrictions (e.g. via import tariffs or quota), domestic prices may rise to well-above international market levels, to the benefit of domestic farmers but making food more expensive for consumers; support for one constituency typically comes at the expense of another (whereas smallholder families may be negatively affected as well in case they are net-buyers of food). Moreover, such policies go against the WTO principles stating gradual liberalization of trade is pursued based on equality and reciprocity as two important pillars, in order to guarantee equal playing field in international trade (www.wto.org).

Most developing countries have room for policy manoeuvring within the internationally agreed WTO framework and trade rules, because most current tariffs fall short of bound tariffs (that is, they are below the upper limits on tariffs; see Matthews, 2014; Laroche Dupraz & Postolle, 2016). For many less economically developed countries, import tariffs are usually the only policy tool available, since they can hardly afford to subsidise their farmers.

However, as noted above, raising tariffs can generate significant costs and will not necessarily improve food security or reduce consumer prices or facilitate trade flows. Nevertheless, tariff hikes—if only temporary—may be worth considering there is a trade-off between using limited public resources for agricultural subsidies vs using them to invest in rural infrastructure, education and social protection.

When the 1995 WTO Agreement on Agriculture (AoA) set spending ceilings on agricultural support, it distinguished between price and income support measures. To date, developing countries scarcely use “more than minimally” trade-distorting domestic subsidies below what is permitted by the AoA (Matthews, 2014). In addition, developing countries in pursuing their food security goals are entitled to unrestricted use of domestic funding for:

- “green box”—government-funded direct payments to farmers for the delivery of environmental services, that are assumed not to distort trade (WTO Annex 2), and for
- investment subsidies to support innovation and competitiveness generally available to agriculture in developing country members, and agricultural input subsidies generally available to low-income or resource-poor producers (WTO Article 2).

Assuming that financial sums spent under the agricultural support practices will not “more than minimally” affect other countries’ production and trade, developing countries should consider using the investment and input subsidies allowed under WTO rules to the greatest extent possible but with a significantly different format than current subsidies which often decrease overall economic efficiency, lead to over production and create perverse health, environmental and equity outcomes. For the most fragile poor countries, if they enact tariffs to protect their agriculture (as explained above), the revenues from those tariffs could help fund agricultural sector subsidies. However there is a trade-off between using limited public resources for agricultural subsidies versus using them to invest in rural infrastructure, education and social protection. Related to this are complex questions of targeting and who really benefits from subsidy payments.

### Adopt trade and market facilitation policies to build a competitive food sector

To make the best use of export market opportunities, governments can align SPS (Sanitary and Phytosanitary) measures—and other non-tariff measures (NTMs) affecting trade—with regional standards and global (WTO) standards. As cross-border movements of foods continue to increase, the potential for contaminant spread is high, prompting a global focus on safety and quality. The WTO SPS Agreement sets out the basic rules for food safety and animal and plant health standards. The Technical Barriers to Trade Agreement (TBT) concerns standards and technical regulation in areas other than health and safety: these areas include quality, environment and social welfare.

Many countries aspiring to enter global agri-food trade have a critical need for international assistance with food safety and quality investments. Because trade in agri-food products is increasingly affected by domestic food safety and quality regulations of destination countries, investing in these areas is a precondition for benefiting from such trade (OECD, 2020; OECD, 2019a, b). Setting up and managing a food safety system is a broad challenge: it encompasses regulations, infrastructure such as laboratories, cold storage facilities, management systems and ICT networks, and requires risk-assessment organizations such as inspection services and accreditation bodies. Many developing countries lack the human capacity and resources to set up such a system in accordance with international standards (AGRA, 2020; Duval et al., 2018).

Investment in trade facilitation policies is key to reaping the benefits of trade: these mainly concern customs procedures, taxes, permits, and administrative trade costs. Poor trade facilitation is a significant driver of food insecurity in Africa, where inter-regional trade suffers greatly under complex and burdensome import and export procedures. Food availability and food access are significantly reduced by higher documentation requirements and long export and import times (Bonuedi et al., 2020). The most effective trade facilitation reforms to increase food security in Africa are those that reduce delays caused by documentary and border compliance procedures. In particular, infrastructure improvements and digitized trade procedures reduce trade costs significantly (see also Duval et al., 2018; for examples in Asia-Pacific).

## Standards compliance conditional to fully realize benefits from trade

Public and private standards, spread through trade and foreign direct investment (FDI), are increasingly important for regulating international trade (Swinnen, 2016; Swinnen & Kuijpers, 2020). To enter and benefit from these markets, low income countries must invest in efforts to raise domestic production and consumption standards, and in programs to reinforce compliance. Including smallholder farmers in food value chains subject to international standards poses multiple challenges, since poor farmers lack the resources necessary to invest in standards compliance, and local institutions are not equipped to guarantee surveillance. This means that innovative strategies for involving key stakeholders in the design, implementation and compliance of food (safety and quality) standards are required.

In recent years, developing countries in Africa and Asia have achieved strong growth in sectors with rapidly spreading standards. Examples include high value food products such as fruits, vegetables, seafood, fish, poultry and dairy products. These standards support food exports, but also contributed to domestic food market upgrading.

Standards can thus promote trade but not necessarily support inclusive food markets. Different factors influence how the gains from such trade are divided between domestic or foreign populations, and between consumers or producers. This depends on particular aspects of the standard (i.e. whether it includes product attributes related to safety, quality and health, or also other attributes related to production systems, such as fairness or sustainability) and on how these aspects are implemented (public, private or voluntary) (Swinnen, 2016).

Empirical literature shows that exporting traders and firms frequently applied contract systems, including technology transfers and provisions of inputs, to ensure that farmers can comply with food safety, quality and other specific standards. For instance, Minten et al. (2009) find that access to technological inputs motivated smallholder vegetable farmers in Ethiopia to sign contracts with exporting companies. Bellamara and Novak (2016) show that in other African value chains, such as those for cotton, rice and barley, contract systems with extensive inputs and technology transfers are common for exporters and processors. Describing the growth of high-value agriculture in Asia, with examples from India, Bangladesh, Pakistan, Thailand, Vietnam, Indonesia and the Philippines, Gulati et al. (2007) identify important positive effects on farmers’ productivity from the rapid rise of their vertical linkages with retailers, processors and traders and exporters in various forms of contract farming. These forms include input provisions and technology and knowledge transfers.

While most pertinent studies focus on export supply chains, some have looked at contract farming systems within chains with mostly domestic operations (e.g. Swinnen, 2007; Berkum, 2007; Dries et al., 2009 for examples in value chain contracting systems in Eastern Europe and Central Asia; Van Campenhout et al., 2019 for an example in Uganda’s dairy sector). Empirical literature shows that local smallholder suppliers—with limited access to capital and technology—can be integrated with high value high standard sectors through value chain governance based on contracting and on hybrid forms of vertical integration involving technology and input transfers (Swinnen & Kuijpers, 2020; Ton et al., 2017).

When smallholder farmers participate in high-standard export production and trade, their participation does not always improve rural livelihoods and reduce poverty. This also depends on how attractive or necessary farmers’ involvement appears to traders or processors. Smallholders are more likely to participate in value chains when the farm sector is more homogeneous and when the region contains mostly small scale farms (Vandemoortele et al., 2012). In contrast, when local production structures are more mixed, sourcing from smallholders only occurs when it is not more expensive than sourcing from large farms.

Policies to enhance smallholders’ integration into supply chains focus on reducing transaction costs for smaller and less resourceful producers for entering more modern value chains. Such policies include, for example, managing foreign direct investment (FDI) so as to integrate smallholders, investing in rural infrastructure (roads, storage facilities, energy, ICT networks) to connect small-scale farmers in remote areas with markets. Moreover, farmers need to be empowered to obtain a better bargaining position in the supply chain. Government policies may support the establishment of producer organizations with proper legislation, with information and knowledge transfers enabling them to operate such organizations, sometimes using financial support measures (such as tax exemptions). Also helpful for integrating smallholders into value chains are policies that invest in institutions for independent quality and food safety control, certification, public extension and market information services (Swinnen & Kuijpers, 2020; Ton et al., 2017; Reardon & Timmer, 2012).

## Trade policies need to incorporate externalities and to reinforce non-market values

Improving the environmental and nutritional impacts of food systems is a key objective of food system transformation, and the management of food trade plays a major role in achieving this. Current trade systems focus on market values and economic efficiency, failing to integrate externalities into market prices. To support environmentally sustainable, nutritionally dense and safe food systems, a global system of trade arrangements that recognizes these non-market values is needed. A vital condition of success is that contracts and regulations intended to protect non-market values are incorporated into domestic food systems.

### Environmental challenges

Agricultural trade can have in certain cases advantageous effects on the environment, for instance by saving water resources from trade (that is, the exporter of the commodity uses less water that the importer would consume if it produced the food domestically (Dalin & Rodriquez-Iturbe, 2016; Balogh & Jambor, 2020). At the same time, expansion of trade may facilitate specialisation in the exporting country and induce greater reliance on more input-intensive production methods, which can leave detrimental environmental effects on soil degradation, nutrient depletion, deforestation, erosion, water logging and climate change (Balogh & Jambor, 2020). In recent decades, significant deforestation in the Amazon biome, in some Southeast Asian countries and in some African countries—such as Angola and Zambia—have added to global GHG emissions and biodiversity loss. Deforestation is known to be driven partly by international trade (Dalin & Rodriquez-Iturbe, 2016; Pendrill et al., 2019)

Efforts to make tropical agriculture more sustainable and to slow down deforestation have confirmed the vital importance of governance actors, including private sector and societal non-governmental organizations, and of technical monitoring capacity (DeFries et al., 2013; Carodenuto, 2019; Arts et al., 2019; FAO, 2020).

The literature on the environmental effects of agricultural trade suggests three categories of solutions to address trade-related negative environmental externalities (Balogh & Jambor, 2020):

- Consumers (mainly in developed countries) should be incentivized to reduce consumption of livestock products (specifically beef), because demand for these products is an important factor in the trade-environment nexus (e.g., Poore & Nemecek, 2018; Duku et al., 2020);
- Environmental harm can be reduced or mitigated by adopting sustainable technologies (i.e. precision agriculture, drought-resistant seeds) and improved natural resource management practices (for nutrients, pests, water and soil management)—both of which require investments in knowledge and technologies for the agricultural sector, the costs of which can only be recouped by producers if consumers or government/society actually pay for them.
- Trade-related policies and regulations can contribute to limiting environmental degradation. Such agreements must be harmonized at the international level, not only for environmental reasons but also to reduce compliance costs for exporters. While environmental provisions have increasingly figured in regional trade agreements (OECD, 2020), they generally lack specific environmental targets.

To better integrate sustainable production standards into trade agreements, exporting and importing countries will need to embrace more commonly established sustainability standards, declare these standards binding and include these in bilateral or regional trade agreements. This can also be a reason to seek for policy space within the WTO multilateral trade context for sustainable and inclusive production methods, especially when environmental costs of production can be assigned monetary values (see e.g. Aspenson, 2020 and TEEBAgrifood, 2019 for examples of true costs accounting methods of agricultural production). It is clear that the inclusion of sustainability criteria in trade agreements and the translation of environmental costs into prices requires collective action on a global scale. Without such a joint effort, there is no level playing field necessary for lasting trade relationships that both importing and exporting parties can benefit from.

### Food safety and nutrition challenges

Trade rules do not generally include objectives for the provision of healthy diets. To improve nutrition outcomes through trade agreements and instruments, developing countries can currently only frame and adopt trade-compliant policies that look at sanitary and phytosanitary standards (for which the WTO SPS Agreement refers to the joint FAO/WHO Codex Alimentarius as the relevant standard-setting organization) and support safe food without discriminating against either domestic or foreign products.

Trade can contribute to protect consumer safety and promote healthy diets only if the standards and regulations applied to food trade are reflected in domestic food systems (Global Panel, 2020). To counter allegations of disguised protection, transparent measures are needed. The necessity of interventions must be clear—and a comprehensive approach must incorporate both imported and domestically produced products, ensuring that policy measures do not discriminate. Informal traditional markets where most poor people buy their fresh and nutritious foods (e.g. eggs, green leafy vegetables and fish) are the major source of safety and health risk (Grace, 2015). Formal regulation is difficult to enforce and better results are reached with broader interventions in clean water and sanitation combined with awareness-raising amongst producers and value chain participants.

### Living wage and social inclusion

In response to civil society concerns in (mainly) importing developed countries, voluntary certification schemes emerged that offer a price premium when achieving more sustainable and social practices. Results in terms of income, inclusion and environmental effects are, however, mixed and successes highly context specific (Ruben, 2020; Waarts et al., 2021). In their case studies of certification schemes in the banana and cocoa sector in respectively Costa Rica and Cote d’Ivoire Alho et al. (2021) find only modest benefits to workers’ livelihood. In Costa Rica, the extent to which certification schemes were responsible for addressing the living wage gap is unclear also because their benefits are commonly correlated with environmental improvements and reduction in pesticide usage. The impacts of premiums paid by certification schemes for income of cocoa smallholders in Cote d’Ivoire proved to negligible, and that the average premium paid is insufficient to raise income to a ‘living income’ level.

Additional measures are thus required if prices are increased to achieve living incomes for smallholders and to ensure no negative effects materialize. Such additional measures need to tackle key bottlenecks of adopting farm management practices necessary to engage and be successful in competitive markets, which are among others improved access to good quality inputs, credits and extension, and a sound business environment that helps farmers manage production, financial and legal risks.

### Incorporating externalities into food prices: trade-offs or synergies?

Food production involves environmental and diet-related health costs that are not currently factored into prices. But if they were, agricultural production costs and food prices would probably be higher. Hence, there is potentially a tension between incorporating externalities in food prices and keeping food affordable, especially to the poor. Moreover, complying with environmental regulations aiming at reducing environmental degradation most likely adds to farmers’ production costs which, if these are not paid for by consumers, might reduce farmers’ profit margins and income. How to go about these potential trade-offs?

A range of economic tools for cost internalization in the agricultural sector has been developed over the decades, from payment for ecosystem services to tax and subsidy programs. Also, voluntary market-driven certification schemes in the agricultural sector are also widely recognized mechanisms through which external environmental impacts associated with agricultural production can be internalized into the price of food; some schemes have objectives to pay smallholder farmers ‘fair’ prices, sufficient for achieving a ‘living income’ (Waarts et al., 2021). However, generally environmental and health costs are currently hardly incorporated in agricultural prices or via direct payment measures, mainly because the emphasis in the market and trade model is on economic efficiency (Clapp, 2017).

Now that environmental sustainability and nutritious food are being embraced on a larger scale as a desired food system outcome, trade rules need to shift focus as well. This requires more policy space for environmental protection and healthy food in trade agreements. It can also mean restricting open trade at the expense of stress on water resources, deforestation or greenhouse gas emissions above country commitments for reduction. Such a rethinking of the contribution that trade can make to sustainable and inclusive agriculture requires a reappraisal of the function of agriculture that goes beyond ensuring tradable products (namely providing essential ecological services, culture and livelihoods, among others).

More attention to ecological costs of production could lead to higher prices for food (that is, will not be countered by ‘ more sustainable’ technology or practices). This in turn can be a major problem for the poorer part of the population. For the most vulnerable population group, social safety net and targeted food programs (i.e. conditional cash transfers, nutritional programs for women and youth, school lunch programs, food-for-work programs etc.) are more effective instruments to improve access to affordable food (Díaz-Bonilla, 2017). As factoring in ecological costs will raise food prices for everyone, increased income and better (non)farm employment opportunities remains the best way of enhancing food security.

## Conclusions

Trade in agricultural commodities and food is important to support domestic availability of food, but it can only contribute to food security if food access, affordability of food and stable food supplies are also guaranteed. The potential contributions of trade to processes of food system transformation have a wider significance: it looks at food trade as a vehicle for reducing shocks and improving food quality and safety, it considers the competitiveness on food markets as a core dimension for reducing rents and for supporting inclusiveness, and it addresses the governance and level-playing field for public regulation and private compliance of food standards.

Although trade has clearly increased food availability in global aggregate, it can also pose threats to food security for particular countries and populations. Trade can increase dependency on food imports and lead to indebtedness, it can also make food supplies more vulnerable and threaten the competitiveness of smallholder farmers. Since much of the current trade systems focus on market value and economic efficiency, they fail to integrate social and environmental externalities into market prices—a failure that harms the environment and makes diets less healthy.

To address these trade-offs between trade openness and desired food system outcomes policy strategies should focus on diversifying production and markets, and on improving the sector’s competitiveness. Next, for small-scale farmers to participate in modern value chains and benefit from trade, they need access to inputs and technologies, and support to comply with grades and standards. And finally, to support environmentally sustainable, nutritionally dense and safe food systems, a global system of trade arrangements that recognizes these non-market values is needed. A vital condition of success is that contracts and regulations intended to protect non-market values are incorporated into domestic food systems.

## Footnotes

1 Examples of unilaterally determined non-reciprocal preferential trade schemes are the Caribbean Basin Initiative (CBI), promulgated by the United States in favour of 17 Central America and Caribbean countries and territories washed by the Caribbean Sea, and the EU’s General System of Preference (GSP) applied to low and lower-middle income countries (GSP standard and GSP+), and to the least developed countries (Everything But Arms, EBA).
